# The Most Common Lesions Detected by Neuroimaging as Causes of Epilepsy

**DOI:** 10.3390/medicina57030294

**Published:** 2021-03-22

**Authors:** Bożena Adamczyk, Karolina Węgrzyn, Tomasz Wilczyński, Justyna Maciarz, Natalia Morawiec, Monika Adamczyk-Sowa

**Affiliations:** Department of Neurology in Zabrze, Faculty of Medical Sciences in Zabrze, Medical University of Silesia Katowice, 40-055 Katowice, Poland; karolinwegrzyn@gmail.com (K.W.); tewik94@gmail.com (T.W.); pique1nique@gmail.com (J.M.); nataliamorawiec007@gmail.com (N.M.); m.adamczyk.sowa@gmail.com (M.A.-S.)

**Keywords:** neuroimaging examinations, epilepsy, hippocampal sclerosis, focal cortical dysplasia, tumours, vascular malformations

## Abstract

Epilepsy is a common neurological disorder characterized by chronic, unprovoked and recurrent seizures, which are the result of rapid and excessive bioelectric discharges in nerve cells. Neuroimaging is used to detect underlying structural abnormalities which may be associated with epilepsy. This paper reviews the most common abnormalities, such as hippocampal sclerosis, malformations of cortical development and vascular malformation, detected by neuroimaging in patients with epilepsy to help understand the correlation between these changes and the course, treatment and prognosis of epilepsy. Magnetic resonance imaging (MRI) reveals structural changes in the brain which are described in this review. Recent studies indicate the usefulness of additional imaging techniques. The use of fluorodeoxyglucose positron emission tomography (FDG-PET) improves surgical outcomes in MRI-negative cases of focal cortical dysplasia. Some techniques, such as quantitative image analysis, magnetic resonance spectroscopy (MRS), functional MRI (fMRI), diffusion tensor imaging (DTI) and fibre tract reconstruction, can detect small malformations—which means that some of the epilepsies can be treated surgically. Quantitative susceptibility mapping may become the method of choice in vascular malformations. Neuroimaging determines appropriate diagnosis and treatment and helps to predict prognosis.

## 1. Introduction

Epilepsy is one of the most common neurologic disorders, affecting approximately 1% of the world population. The detailed pathophysiology is still not completely understood. It can have numerous causes that reflect underlying brain dysfunction [1]. In approximately two-thirds of cases, the disease begins during childhood [1]. Despite the constant development of diagnostic and therapeutic tools, drug therapy fails to control epileptic seizures in about one-third of patients [2]. Uncontrolled seizures can cause a significant decrease in the quality of life of patients. Therefore, proper diagnosis and therapy are essential.

Structural abnormalities which may be associated with epilepsy can be detected using neuroimaging. Surgery is the preferred approach in some cases which are refractory to pharmacotherapy [3]. In these patients, the location of the epileptogenic focus plays a key role in successful neurosurgery as the outcome depends on complete resection, ablation or disconnection of the epileptogenic zone [3].

The present paper reviews the most common abnormalities detected in neuroimaging of patients with epilepsy. Special attention was paid to the relationship between these changes and the course, treatment and prognosis of epilepsy in such patients.

## 2. The Most Common Imaging Abnormalities

The common brain structural abnormalities associated with epilepsy are as follows: hippocampal sclerosis, malformations of cortical development (MCD), tuberous sclerosis complex, low-grade gliomas, vascular malformation and hypothalamic hamartoma [4]. Rasmussen’s encephalitis is less frequently observed [5] (Table 1). 

### 2.1. Hippocampal Sclerosis

Hippocampal sclerosis (HS) is the most common abnormality among patients with temporal lobe epilepsy (TLE), especially mesial temporal lobe epilepsy (MTLE) which tends to be refractory to antiseizure medications (ASMs) and is the most prevalent type of the epileptic syndrome [6,7,8,9,10,11]. Magnetic resonance imaging (MRI) is the best method to visualise HS, which is characterized by the loss of internal structures, decreased volume, decreased signal on T1-weighted images and increased signal on T2-weighted fluid-attenuated inversion recovery (FLAIR) MRI images [12,13].

The use of coronal slices of 3-mm thickness is optimal for detailed neuroimaging of the hippocampus. However, slices <3-mm thickness would be preferred [14].

Using 3-Tesla MRI (3T MRI) instead of 1.5-Tesla MRI (1.5T MRI) can improve visualization of abnormalities in the internal hippocampal architecture at earlier stages of the disease, which are seen as neuronal loss, neuronal fibre loss and gliosis [9].

The comparison of the utility of 3T vs. 1.5T MRI is difficult because imaging tests using different Tesla values are rarely performed in short time intervals. Scientists did not note any significant differences in neuroimaging, which had an important influence on the diagnosis of HS [15]. Nonetheless, they reported that 3T MRI revealed HS abnormalities, which are not detectable on 1.5T MRI. Thus, for many patients with HS, 3T MRI seems to be superior. In some cases, like dual pathology of cortical dysplasia along with hippocampal sclerosis, 1.5T MRI may be more useful. [16].

There were some reports on high-quality imaging with 7-Tesla MRI which showed a high correlation with histopathological changes in the hippocampus. This seems to be a significant predictor for specifying the type of HS in TLE patients undergoing radical treatment, as well as their clinical outcome [17].

Recent reports describe a study in which 4.7-Tesla MRI was applied for visualization of the whole hippocampus using a T2-weighted (inverted contrast) fast-spin-echo technique. Acquiring 90 contiguous coronal slices of 1-mm thickness enabled precise calculation of the volume and effectively differentiated HS subtypes [18].

The analysis of other structures of the limbic system can provide information related to HS. One study based on the thin-slice-reconstructed volumetric analysis (thickness of 0.2 mm) showed atrophy of the mammillary body on the same side of HS, which conventional neuroimaging does not reveal [19].

In their study, Singh et al. showed MRI-negative patients with an enlarged amygdala, which may be suggestive of MTLE [20]. However, Nakayama et al. showed that the examination of amygdala adjacent to the hippocampus may not provide relevant information due to the lack of parallel changes in HS with respect to volume loss in the amygdala [21].

Surgical treatment should be considered immediately in patients with intractable MTLE-HS. A delay in surgery can result in poorer outcomes and escalation of cognitive disorders [14,22,23]. Studies revealed the superiority of surgery over ASMs therapy in MTLE-HS patients who did not respond to drug therapy. Postsurgical seizure-freedom rates ranged from 60–70% after 1–2 years of follow-up to 40–50% after 10 years of follow-up. With ASMs treatment, follow-up after an average of 27.3 years showed that 29% of patients were seizure-free [12,13,14].

### 2.2. Malformations of Cortical Development

Malformations of cortical development (MCD) are cortical lesions due to abnormal intrauterine developmental processes and contain cells responsible for the cortical mantle formation [24]. MRI remains one of the most useful techniques in the case of MCD. Fluorodeoxyglucose positron emission tomography (FDG-PET) is a key diagnostic method for MRI-negative cases [25].

Other imaging techniques, including quantitative image analysis, magnetic resonance spectroscopy (MRS), functional magnetic resonance imaging (fMRI), diffusion tensor imaging (DTI) and fibre tract reconstruction, can detect small malformations. Long-term video-electroencephalography can be used to confirm the diagnosis of epilepsy and to help to localize the epileptogenic zone [26].

Thus, some of the epilepsies previously defined as cryptogenic can be treated surgically [27].

#### 2.2.1. Focal Cortical Dysplasia

Focal cortical dysplasia (FCD) is one of the most common malformations with a prevalence of 48% among patients diagnosed with epilepsy [28]. It is characterized by disorganization of the cortical lamination related to dysplastic neurons or increased volume of cells with eosinophilic cytoplasm [29]. Although 25–33% of patients with FCD (most often within the first year of treatment) respond to antiepileptic medication [30], treatment is mainly surgical and depends on the identification of structural and functional lesions [31]. Thickening or pseudothickening of the grey matter, blurring of the grey-white junction, increased T2 signal in the white matter and structural atrophy are found in MRI. No seizures are experienced in 80% of patients following total resection of the cortex, as opposed to 20–50% in patients with incomplete resections due to a lack of imaging findings or involvement of the eloquent cortex [28]. Using FDG-PET was a significant step toward solving the problem of detecting FCD in MRI-negative cases. Although accuracy is not always 100%, it has improved surgical outcomes [30].

Magnetoencephalography (MEG) is an additional technique used to localize the epileptogenic zone [28] in which brain activity is mapped by recording magnetic fields generated by neuronal activity, using magnetometers. Thereby, the pathological region can be detected. It is approximately equally sensitive to MRI [28,32].

Electrical source imaging (ESI) is another method that may change the surgical management of FCD patients with drug-refractory epilepsy. The epileptogenic zone can be precisely localized with ESI on intracranial EEG. This can help with surgical planning and predicting the outcomes of epilepsy surgeries [33].

#### 2.2.2. Polymicrogyria

Polymicrogyria (PMG) is characterized by developmental abnormalities where cortical neurons reach the cerebral cortex. However, neurons are distributed abnormally, resulting in abnormally small gyri. PMG is an important contributor to medically refractory epilepsy [34]. It is usually associated with a grey matter heterotopia, ventriculomegaly and abnormalities of the white matter, corpus callosum, brain stem and cerebellum. Because of high resolution and adequate contrast, MRI is the recommended method to identify the small folds that define PMG. With high-quality MRI, microgyri and microsulci may be appreciated and specific stippling of the grey-white junction may be observed. The assessment of Sylvian fissures is crucial, because PMG often affects these areas preferentially [35]. Treatment of epilepsy associated with PMG is typically ASMs, however, surgery is considered in drug-refractory cases [34].

#### 2.2.3. Grey Matter Heterotopia

Grey matter heterotopias (GMHs) are a group of neurological disorders caused by conglomerates of grey matter along the ventricular walls or in the white matter. GMHs can be divided macroscopically into: nodular heterotopias (subependymal, subcortical) and diffuse heterotopias (lissencephaly, laminar and band heterotopia) [36]. They are an important cause of epilepsy. Large lesions may be detectable in CT (computed tomography) because of their slightly higher density than the surrounding white matter. On MRI, the heterotopic tissue can be seen as grey matter with indistinct margins on all sequences [37].

#### 2.2.4. Periventricular Nodular Heterotopia

Periventricular nodular heterotopia consists of conglomerates of apparently normal brain cells located periventricularly or subcortically and results from impaired neuronal migration [38]. It can cause refractory epilepsy and, in such cases, surgery should be considered. However, it was reported that the outcome is usually unsatisfactory in patients with bilateral lesions. Selective stereotactic ablation with radiofrequency thermocoagulation (RFTC) guided by stereoelectroencephalography (SEEG) was proposed as an alternative option in these patients [39].

MRI is a commonly used neuroimaging method in periventricular nodular heterotopia. On MRI scans, multilobar cortical thickening encompassing prefrontal, latero-basal temporal and temporoparietal cortices may be seen [40]. Resting-state fMRI enables the detection of areas of abnormal function. It is the best method to identify the resting state networks [41].

FDG-PET can be useful, especially in surgical planning. Combined FDG-PET/MRI imaging may improve the identification of epileptogenic foci and result in postoperative seizure control [42].

#### 2.2.5. Hemimegalencephaly

Hemimegalencephaly is an overgrowth of one cerebral hemisphere. It is connected with the triad of epilepsy, global developmental delay and contralateral motor deficit. Patients have a poor neurodevelopmental outcome and epilepsy is difficult to treat [43]. MRI is the best method to diagnose hemimegalencephaly. It shows asymmetric enlargement of one cerebral hemisphere and diffuse thickening of cortical grey matter with increased myelination over the enlarged hemisphere [44]. There is usually no consistent preference for which lobe of the brain is affected by hemimegalencephaly. It is often associated with enlargement of ipsilateral ventricle and neuronal heterotopias [45]. In FLAIR MRI, white-matter-signal intensity may change [46]. Hemispherectomy is often performed as a method of reducing the number of seizures. The results are unclear because the outcomes of children have rarely been studied into adulthood [43].

### 2.3. Tuberous Sclerosis Complex

Tuberous sclerosis complex (TSC) is a neurocutaneous, autosomal-dominant syndrome that can present at any age. It may affect multiple organ systems by evolving hamartomas in skin, the central nervous system, kidneys, lungs or heart [47]. In almost 30% of cases, the occurrence of a triad of symptoms are characteristic: seizures, intellectual disability and cutaneous angiofibromas [48]. Brain malformations that contribute to the development of epileptic seizures may be visualized by MRI. The detected lesions include cortical tubers, radial migration lines, subependymal nodules and subependymal giant cell astrocytomas [49]. In infants, cortical tubers appear as hyperintensive lesions on T1-weighted images and hypointensive on T2-weighted images. Then, after myelin maturation, only T2-weighted scans show thickened gyri, poor grey-white matter differentiation and a subcortical (“flame-shaped”) hyperintensity. Radial migration lines are mildly hyperintense on T1-weighted images in infants. After myelination they are hyperintense on T2-weighted sequences and can be moderately enhanced. Subependymal nodules, because of calcified components, are hyperintensive both on T1- and T2-weighted scans. Subependymal giant cell astrocytoma is an intraventricular growing mass with mixed T1-/T2-weighted signal and intense contrast enhancement. For differential diagnosis from large subependymal nodules, MRS is useful [50]. In surgical planning, nuclear medicine techniques (α-[11C]-methyl-l-tryptophan positron emission tomography (AMT-PET), FDG-PET and single-photon emission computed tomography (SPECT)) may be useful to differentiate epileptogenic zones from nonepileptogenic lesions [50].

### 2.4. Low-Grade Gliomas

Low-grade gliomas (LGGs) are most commonly diagnosed among adults in the fourth decade of life. They include diffuse astrocytomas and oligodendrogliomas [51]. In more than 90% of patients, epileptic seizures occur. Therefore, these lesions are highly epileptogenic [52]. MRI characteristics include low-intensity on precontrast T1 and enhancement after contrast administration, as well as high-intensity on T2 and FLAIR (excluding oedema) [53]. To distinguish low-grade from high-grade gliomas, amide proton transfer-weighted MRI may be performed [54]. In differentiation, 18F-FACBC (synthetic L-leucine analogue-based) PET also may be helpful [53].

### 2.5. Dysembryoplastic Neuroepithelial Tumours

Dysembryoplastic neuroepithelial tumours (DNETs) are benign glioneuronal tumours which are characterized by the early onset of seizures [55] .These highly epileptogenic lesions are usually located in the cerebral cortex, especially in the mesial temporal lobe. DNETs often coexist with focal cortical dysplasia, hippocampal sclerosis and ganglioglioma [56]. Typically, they are well-demarcated structures which are hypointensive on T1-weighted and hyperintensive on both T2-weighted and FLAIR MRI. Cysts and microcalcifications are characteristic for those lesions [57]. Usually, there is no mass effect or oedema. To distinguish DNETs from other low-grade gliomas, MRI FLAIR and MRS are recommended. In FLAIR MRI, internal septation and characteristic “ring sign” on the periphery may be revealed. In MRS there is a low N-acetylaspartate peak and a lack of elevated choline-containing component or Cho-Cr ratio [57,58]. The preferred method of DNET treatment is total tumour resection, which usually results in the stabilization of epileptic seizures [58].

### 2.6. Vascular Malformations

It was reported that 11% of all adult epilepsy cases and 45% of epilepsy cases in patients over 60 years of age are the result of ischaemic or haemorrhagic stroke [59]. Vascular malformations (VMs) and cerebral cavernous malformations (CCMs) are the most common epilepsy-associated vascular lesions [60].

#### 2.6.1. Cerebral Cavernous Malformations

Cerebral cavernous malformations (CCMs), also known as cavernomas, are found in 4% of the general population [60] and consist of dilated, endothelium-lined blood vessels without arterial features. Thrombosis, calcifications and inflammatory changes may be seen [61]. Epilepsy is the most common clinical presentation of CCM (37%) [62] and it is significantly more common than small asymptomatic haemorrhages [63], which result in progressive haemosiderin deposition in the brain, causing chronic irritation and the initiation of the process of remodelling of the cerebral cortex [64]. MRI reveals heterogeneous blood products in both T1- and T2-weighted sequences, depending on the time of haemorrhage. The surrounding oedema is not present unless recent bleeding has occurred [64].

T2-weighted gradient-recalled echo (GRE) or susceptibility-weighted imaging (SWI) can enhance the detection of calcifications, haemorrhages and small vascular malformations [65]. Acquisition with a coronally oriented T2 GRE sequence improves the detection of small lesions located in the proximity of the skull base.

Quantitative susceptibility mapping (QSM) is a recently developed MRI technique that provides direct and precise quantitative measurement of iron deposition in the brain tissue [66]. It has been proposed that QSM could become a neuroimaging biomarker in CCM lesions, for monitoring the activity of the disease as well as the response to treatment.

#### 2.6.2. Arteriovenous Malformations

Arteriovenous malformations (AVMs) are abnormal blood vessels in the brain with arterial blood flowing directly into draining veins with no interposed capillary bed. As a result of haemorrhage, seizures are the most common presenting symptom in 30% of patients [67]. AVMs which present with an intracranial haemorrhage or a focal neurologic deficit correlate with a higher seizure risk compared to incidental AVMs [68].

Perinidal hypoxaemia, venous congestion, long pial vein and a space-occupying effect are among angiographic features related to epileptic seizures [69]. MRI shows vessels with a typical appearance of a “bag of black worms” (flow-void structures) with minimal or no mass effect. High resolution T2-weighted fast-spin echo is the best sequence for visualizing AVM features. Flow visualization can be best examined using three-dimensional time-of-flight MR angiography (3D TOF MRA), whereas time-resolved MR angiography (TR-MRA) with contrast enhancement may be used additionally to visualize the angioarchitecture [70]. Treatment is still controversial and conservative management is favoured in asymptomatic patients.

### 2.7. Hypothalamic Hamartoma

Hypothalamic hamartoma (HH) is a benign congenital malformation of the hypothalamus that develops in foetal life [71]. Considering the location of the lesion, two subtypes are distinguished: parahypothalamic and intrahypothalamic hamartoma. Epilepsy and neurobehavioral symptoms, as well as central precocious puberty and developmental delay, are the main clinical symptoms of HH. The gold standard in the diagnosis of HH is MRI. A decreased intensity in T1-weighted scans and an increased intensity in T2-weighted scans are observed. Lesions do not enhance after contrast administration. HH usually coexists with the presence of cysts, especially arachnoid cysts [72]. Epileptic seizures are drug-refractory, and therefore surgical methods are of interest. Alternatively, due to the difficulty of surgical access, stereotactically targeted radiation therapy or radiofrequency lesioning are used. However, noninvasive methods are preferred [73].

### 2.8. Rasmussen’s Encephalitis

Rasmussen’s encephalitis is typically a chronic inflammation of one brain hemisphere in children. It is characterized by progressive unilateral hemispheric atrophy, coexisting neuropsychological dysfunction and intractable seizures, mostly epilepsia partialis continua (EPC) [63]. Histopathological evaluation shows a cytotoxic T-cell reaction against neurons and astrocytes, provoking their apoptotic death. FDG-PET and ictal single-photon emission computed tomography (SPECT) can demonstrate diffuse unilateral hypometabolism and hypoperfusion even before the abnormalities are visible on MRI. On MRI, dissemination of inflammatory lesions over the affected hemisphere may be revealed [60]. A characteristic course from an increased volume and T2-FLAIR signal to a normal signal in the final stage of atrophy can be observed. Initial MRI examinations in most patients reveal unilateral enlargement of the inner and outer CSF spaces. Contrast enhancement is not present. Progressive tissue loss of the ipsilateral hemisphere is detected on follow-up MRI and it is most intensive within the first 12 months following onset. Response to immunotherapy, ASMs, steroids, antiviral drugs and plasmapheresis seems to be poor and temporary. Surgical treatment provides long-term seizure freedom in 80% of patients [60]. However, major postoperative deficits are inevitable.

## 3. Peri-Ictal Imaging Abnormalities

In some patients, neuroimaging methods may reveal peri-ictal abnormalities. The most common changes include restricted diffusion on DWI (diffusion-weighted imaging) and hyperintensity in T2-weighted MRI scans [74,75]. The mesial temporal structures and neocortex are usually affected locations. Changes may be irreversible and are not only localised to the epileptogenic focus [76]. These acute ictal imaging changes are very important because they give an insight into the dynamic neuronal metabolic environment in the peri-ictal period.

Table 2 presents the most common clinical features of epilepsy-related disorders.

## 4. Conclusions

MRI is one of the most important methods for showing HS and allowing the subclassification of epilepsy associated with HS, which may play a vital role in prognosis and treatment. 1.5-Tesla magnets are the most commonly used to detect brain structural abnormalities. However, recent research has demonstrated the possibility to improve the efficiency and the quality of neuroimaging by using magnets of higher strength. MTLE-HS is usually refractory to ASMs, which makes surgery an advisable treatment for a significant number of patients with MTLE-HS. MRI is the most common method for detecting different cortical lesions which determine MCD and provides the opportunity to achieve good surgical treatment outcomes. There are also many new methods of imaging which reveal subtle cortical lesions which contribute to the treatment of intractable epilepsy. FDG-PET is a key diagnostic method for MRI-negative cases.

Seizures are the most common presentation in vascular malformations. As haemorrhage and thrombosis are common features of CCM, MRI reveals heterogeneous blood products on both T1- and T2-weighted sequences. However, as QSM provides more precise iron measurement, it may become the method of choice in the near future. AVMs require MR angiography in order to visualize intracranial vessels. In Rasmussen’s encephalitis, apart from obtaining T2-FLAIR MRI images, FDG-PET and SPECT can provide extra information on metabolism and perfusion.

## Figures and Tables

**Table 1:** The most common imaging abnormalities.

Imaging Examination	Application	Diseases
**MRI**	- detection of HS characterized by the loss of internal structures, decreased volume and increased signal in T2-weighted images - useful in the subclassificaction of HS which is crucial for further treatment decision making and has prognostic value - prediction of the approximate duration of seizure-free period after surgery - useful in predicting a clinical course of epilepsy	HS
	- imaging of thickening or pseudothickening of the grey matter	FCD
	- visualization of cortical gyration with an aspect of cortical thickening and shallow sulci and irregularity of the grey-white matter interface	Polymicrogyria
	- visualization of the overgrowth of one cerebral hemisphere to achieve better outcome after surgical treatment	Hemimegencephaly
	- imaging of cortical hyperintense areas to evaluate the risk of PTE - detection of subtle atrophy using advanced volumetric analyses of submillimeter resolution images	PTE
	- gold standard in tumour evaluation and follow-up	Tumour-related epilepsy
**FDG-PET**	- detection of FCD in MRI-negative cases to improve surgical outcome	FCD
**Electrocorticography**	- determination of the lesion extension	FCD
**MEG**	- localization of the epileptogenic zone	FCD
**UHF MRI**	- analysis of different cortical layers based on the amount of iron, white matter fibres or vascular density	FCD
**CT**	- imaging of frontal or temporal lesions to evaluate the risk of PTE	PTE
	- detection of the majority of tumours except some LGGs, calcifications and bone lesions	Tumour-related epilepsy
**MRS**	- visualization of biochemical aspects of brain changes - sensitive method to reveal the axonal injury in the corpus callosum of TBI patients	PTE
	- IDH mutation indicative of LGG - meningiomas: increased Cho and decreased Cr; evaluation of malignant potential	Tumour-related epilepsy
**fMRI**	- provision of data on altered and compensational brain activity due to injury	PTE
**(FLAIR) MRI**	- more sensitive in detecting traumatic lesions and haematomas	PTE
**PET, SPECT**	- useful in guiding long-term therapy by helping to establish patient prognosis	PTE
**DW-MRI**	- differential diagnosis in ring-enhancing lesions	Tumour-related epilepsy
**^19^Met-PET**	- differential diagnosis of nonrapidly progressing tumours e.g., DNETs and LGGs; normal uptake can be indicative of DNET - treatment planning of stereotactic radiotherapy	Tumour-related epilepsy
**MRI T2 GRE, SWI**	- detection of haemorrhage, iron deposition and small vascular malformations in CCM	Vascular malformations
**MRI CSF flow**	- diagnosis of CIM	Vascular malformations
**MRI T2 FLAIR**	- detection of hemispheric atrophy in RE before it is visible on MRI	Vascular malformations
**3D TOF MRA, TR MRA with contrast enhancement**	- visualization of blood flow and angioarchitecture in AVMs	Vascular malformations

HS—hippocampal sclerosis, FCD—focal cortical dysplasia, MRI—magnetic resonance imaging, FDG-PET—fluorodeoxyglucose positron emission tomography, MEG—magnetoencephalography, UHF—ultra-high-field, PTE—post-traumatic epilepsy, CT—computed tomography, MRS—magnetic resonance spectroscopy, TBI—traumatic brain injury, fMRI—functional MRI, FLAIR—fluid-attenuated inversion recovery, PET—positron emission tomography, SPECT—single-photon emission computed tomography, LGG—low-grade glioma, Cho—choline, Cr—creatine, DW-MRI—diffusion-weighted MRI, ^19^Met-PET—[19C]-methionine PET, DNET—dysembryoplastic neuroepithelial tumours, CCM—cerebral cavernous malformations, GRE—gradient-recalled echo sequences, SWI—susceptibility-weighted imaging, CSF—cerebrospinal fluid, CIM—Chiari 1 malformation, RE—Rasmussen’s encephalitis, 3D TOF MRA—tridimensional time-of-flight MR angiography, TR MRA—time-resolved MR angiography, AVMs—arteriovenous malformations.

**Table 2:** Clinical features of epilepsy-related disorders [77,78,79].

Disease	Clinical Features
Hippocampal sclerosis	- seizures - depression - mood disturbances - poor auditory memory - learning disabilities
Malformations of cortical development: Focal cortical dysplasia Polymicrogyria Grey matter heterotopia Periventricular nodular heterotopia Hemimegalencephaly	- seizures - feeding problems - global developmental delay - deficits in social interactions - stereotyped or involuntary movements - autonomic dysregulation - visual and hearing loss - abnormal head size (microcephaly, megalocephaly)
Tuberous sclerosis complex	- seizures - intellectual disability - hypopigmented macules, cutaneous angiofibromas - renal manifestations: hematuria, loss of kidney function - pulmonary manifestations: cough, dyspnea, hemoptysis, pneumothorax - cardiac manifestations: cardiomegaly, murmurs, changes in the blood flow, arrhythmias, nonimmune hydrops - neurological manifestations: autism spectrum, mood disturbances, behavioral changes, aggressiveness, anxiety, depressive mood, sleep disorders, learning difficulties
Low-grade gliomas	- seizures - behavioral changes - visual disturbance - speech difficulties - loss of muscle strength - symptoms associated with increased pressure in the skull: headache, nausea, vomiting and sleepiness
Dysembryoplastic neuroepithelial tumours	- prolonged temporal seizures - headache - papilledema - focal deficits, in the form of focal weakness or numbness
Vascular malformations: Cerebral cavernous malformations Arteriovenous malformations	- seizures - severe headache - vision loss - speaking difficulties - nausea and vomiting - loss of consciousness - weak muscles - intellectual disability
Hypothalamic hamartoma	- seizures - psychiatric symptoms: externalizing behaviors (aggression, rage attacks), extensive reactivity to minor stimuli, anxiety oppositional defiant disorders, conduct disorders, phobias, post-traumatic stress disorder, avoidant disorder, major depression dysthymia - cognitive impairment, learning disabilities
Rasmussen’s encephalitis	- seizures - progressive neurological and cognitive deterioration with unihemispheric brain atrophy - progressive hemiplegia - unilateral movement disorders, including hemi athetosis and hemidystonia

## Data Availability

Not applicable.
